# Nutlin-3a for age-related macular degeneration

**DOI:** 10.18632/aging.204187

**Published:** 2022-07-16

**Authors:** Hyewon Chung, Chaekyu Kim

**Affiliations:** 1 Department of Ophthalmology, Konkuk University School of Medicine, Konkuk University Medical Center, Seoul 05030, Republic of Korea; 2 Fusion Biotechnology, Inc., Ulsan 44919, Republic of Korea

**Keywords:** anti-aging, cellular senescence, regeneration, retina, senolytic

Age-related macular degeneration (AMD) is a commonly occurring progressive neurodegenerative disease of the retinal pigment epithelium (RPE), retina, and choroid and is a predominant cause of vision deterioration. AMD is divided into early, intermediate, and advanced forms. Early/intermediate AMD (dry AMD) is characterized by extracellular deposits, including drusen, pigmentary changes in the retina, and thickening of Bruch’s membrane [1]. Advanced AMD is further categorized into two morphological forms: wet AMD (neovascular AMD, nAMD) and dry or atrophic AMD (geographic atrophy, GA). nAMD, which is characterized by choroidal neovascularization (CNV), accounts for 10-20% of total AMD cases and can be treated with anti-vascular endothelial growth factor (VEGF) therapy. On the other hand, dry AMD accounts for 80–90% of AMD cases, and no FDA-approved treatment is yet available. Therapeutic approaches, including neuroprotection, inflammation suppression and complement inhibition, are currently being investigated in clinical trials [2]. However, due to the pathophysiological complexity and multifactorial nature of dry AMD, including GA, the development of an effective treatment remains elusive.

Cellular senescence, an irreversible type of cell growth arrest, protects against cancer but also contributes to tissue deterioration underlying aging and age-related pathologies [3]. Senescent cells accumulate in tissues with age and adopt a senescence-associated secretory phenotype (SASP). These cells are associated with the pathophysiology of age-related diseases, including osteoarthritis, atherosclerosis, chronic obstructive pulmonary disease, and Alzheimer's disease [4]. Senescent cells are also found in the retina and RPE in older humans and primates. RPE cellular senescence is linked to potential causes of AMD, including oxidative stress, DNA damage, and mitochondrial dysfunction [5]. Nevertheless, the roles of senescent RPE cells in AMD pathogenesis and development/progression *in vivo* have remained unknown until recently.

In this regard, we hypothesized that cellular senescence in the RPE might represent a new therapeutic target for AMD. We created a chemical-induced mouse model that exhibited general features of cellular senescence in the RPE, including increases in SA-β-gal, p53, p21, and p16 expression; relocalization of HMGB1; and triggering of the SASP [6]. In addition, we performed single-cell RNA sequencing–based transcriptome analysis on control and senescent RPE tissues, which revealed increased senescence-associated gene expression and negative regulation of apoptosis, and on a specific cell population [Lee et al., Communications biology, in press]. The phenotypes observed during RPE cellular senescence overlapped with important pathological features of retinas from patients with dry AMD, such as increases in subretinal deposits, alterations in fundus autofluorescence, and thickening of Bruch’s membrane. Taken together, these findings indicate that senescence in the RPE can contribute to retinal degeneration [6].

We further investigated targeting of the senescent RPE. Two types of drugs that selectively affect senescent cells have been developed, i.e., senomorphics (which suppress the SASP) and senolytics (which kill senescent cells). Senomorphics ameliorate chronic tissue inflammation by regulating the release of SASP molecules from senescent cells. Several drugs that improve mitochondrial function and decrease inflammation, such as rapamycin and metformin, have been investigated [7]. However, these agents exert complex biological effects and influence multiple signaling pathways aside from suppressing the SASP; thus, their clinical value in treating neurodegenerative diseases such as AMD remains uncertain [8]. More fundamental and innovative strategies are needed. Eliminating the source of chronic inflammation by removing senescent cells can improve tissue homeostasis and ameliorate several diseases. Similarly, removing senescent RPE cells might attenuate retinal degeneration and increase overall retinal tissue regeneration capacity.

**Figure 1:**
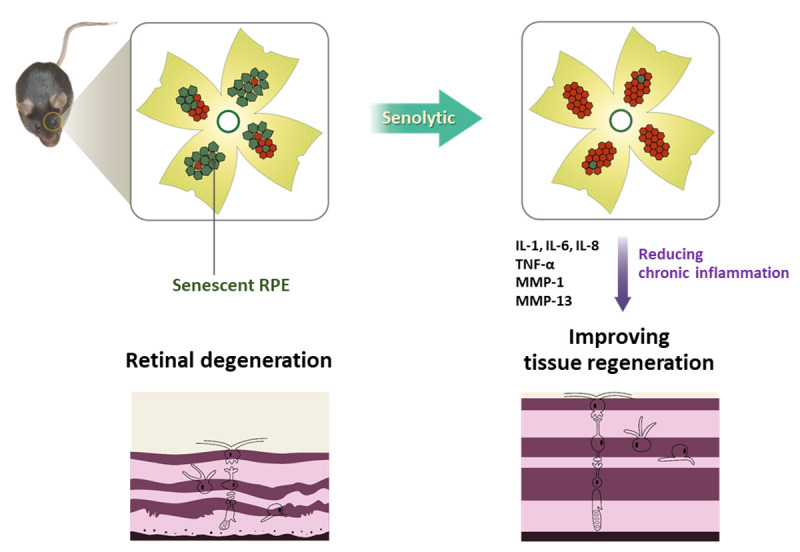
**Elimination of senescent cells improves retinal regeneration.** The MDM2-p53 inhibitor, Nutlin-3a, eliminated senescent retinal pigment epithelial cells and decreased the levels of inflammatory cytokine proteins, ameliorating retinal degeneration in mouse models.

We made an effort and were successful in developing a method to counter the senescent RPE for AMD treatment (Figure 1). We utilized a senolytic drug that selectively induces apoptosis in senescent RPE cells by targeting proteins associated with antiapoptotic pathways [6]. Based on our observation of greater p53 levels in senescent RPE cells than in nonsenescent cells, we hypothesized that an MDM2 antagonist would disrupt the MDM2/p53 protein interaction, increase p53 activity, and trigger p53-dependent apoptosis in senescent cells. The senolytic effect of a potent MDM2/p53 inhibitor, Nutlin-3a, was confirmed in a chemically induced mouse model, an *Alu*-induced GA model, and old mice. Elimination of senescent RPE cells with Nutlin-3a reduced the levels of senescence markers, SASP components, and fundus autofluorescent puncta/deposits. Furthermore, functional evaluation using electroretinograms showed significant recovery of visual function after treatment with Nutlin-3a. Our study suggests that cellular senescence of the RPE might be a key contributor to AMD and thus may be a new therapeutic target for AMD.

There is no approved treatment for dry AMD. Most of the strategies developed to date temporarily impede tissue degeneration by reducing inflammation or complement dysregulation using anti-inflammatory, antioxidant, or antiangiogenic agents. Numerous clinical trials have been conducted on patients with dry AMD, but only a few have obtained partially positive results. Our study provides the first evidence of the use of a senolytic compound as a promising approach for the treatment of dry AMD. Given our findings, further studies are warranted to develop new senolytics for AMD, such as more specific senolytics targeting mitochondria in senescent cells with periodic dosing and longer follow-up. The therapeutic mechanisms of these agents as treatments for AMD should also be investigated.
